# General Slowing and Education Mediate Task Switching Performance Across the Life-Span

**DOI:** 10.3389/fpsyg.2018.00630

**Published:** 2018-05-04

**Authors:** Luca Moretti, Carlo Semenza, Antonino Vallesi

**Affiliations:** ^1^Padova Neuroscience Center, Department of Neuroscience, University of Padova, Padova, Italy; ^2^IRCCS San Camillo Hospital Foundation, Venice, Italy

**Keywords:** task-switching, speed of processing, transparent cue, cognitive aging, cognitive reserve

## Abstract

**Objective:** This study considered the potential role of both protective factors (cognitive reserve, CR) and adverse ones (general slowing) in modulating cognitive flexibility in the adult life-span.

**Method:** Ninety-eight individuals performed a task-switching (TS) paradigm in which we adopted a manipulation concerning the timing between the cue and the target. Working memory demands were minimized by using transparent cues. Additionally, indices of cognitive integrity, depression, processing speed and different CR dimensions were collected and used in linear models accounting for TS performance under the different time constraints.

**Results:** The main results showed similar mixing costs and higher switching costs in older adults, with an overall age-dependent effect of general slowing on these costs. The link between processing speed and TS performance was attenuated when participants had more time to prepare. Among the different CR indices, formal education only was associated with reduced switch costs under time pressure.

**Discussion:** Even though CR is often operationalized as a unitary construct, the present research confirms the benefits of using tools designed to distinguish between different CR dimensions. Furthermore, our results provide empirical support to the assumption that processing speed influence on executive performance depends on time constraints. Finally, it is suggested that whether age differences appear in terms of switch or mixing costs depends on working memory demands (which were low in our tasks with transparent cues).

## Introduction

Many everyday life situations require cognitive flexibility, namely the capacity to adaptively select between multiple task-sets. Task-switching (TS) paradigms are a useful tool for representing such situations within an experimental context. A growing body of research has investigated the underlying cognitive mechanisms of TS, mostly referring to two types of performance costs: the switch and the mixing costs. Switch cost is measured as the difference between a repeat and a switch trial, both in terms of Response Times (RT) and errors, whereas the mixing cost arises as the difference between a repeat and a single-task trial.

The aging literature points to a deterioration in TS performance in the elderly, especially for the mixing cost (Kray and Lindenberger, 2000; Meiran et al., 2001; Adrover-Roig and Barceló, 2010; Lawo et al., 2012). Many accounts of the phenomenon have been put forward, some referring to mechanisms specific for TS (Kray and Lindenberger, 2000; Mayr, 2001) and others trying to integrate age-related difference across different functions, proposing a unitary account of such aging effects (e.g., Lindenberger and Baltes, 1994; Salthouse, 1996; Li and Lindenberger, 1999; Wasylyshyn et al., 2011): in these models, cognitive decline is mainly attributed to deterioration in an underlying domain, rather than considering single domains as separated entities. For this reason, they are referred to as “common cause models”.

One example of these models is the processing speed theory put forward by Salthouse (1996). This model proposes that age-related differences stem from a decline in processing speed, the pace at which simple operations are performed by the cognitive system. Support for this theory mainly comes from studies using hierarchical linear models, or similar ones, to investigate the amount of variance explained by measures of speed of processing, most often consisting of scores obtained in substitution tests; through this type of analyses, similar findings have been replicated for measures of working memory (WM; Salthouse, 1992), Trail Making Test (Salthouse et al., 2000), fluid intelligence (Salthouse et al., 1998), cognitive inhibition (Salthouse and Meinz, 1995; Verhaeghen and De Meersman, 1998; also see Puccioni and Vallesi, 2012a,b), recall, reasoning and spatial abilities (Salthouse, 1993): when the variance explained by speed of processing measures is removed from the models, the relationship of more specific cognitive abilities with age drops down. Importantly for the present study, also the relationship between TS and age was strongly attenuated when controlling for processing speed (Salthouse et al., 1998).

Even though an involvement of speed of processing in determining age-related differences in EFs seems therefore to be likely, it is to be noted that it might not be the only factor at stake (Keys and White, 2000; Verhaeghen et al., 2006; Bugg et al., 2007). Some studies on the Stroop effect, which is consistently found to be increased in older adults, still showed a significant effect of age after controlling for general slowing (Salthouse and Meinz, 1995; Bugg et al., 2007); similar results were also obtained with a card sorting test (Bugg et al., 2007), another task presumed to rely on inhibition and set shifting. Coming to TS, a meta-analysis by Verhaeghen and Cerella (2002) confirmed an effect of age beyond the general slowing, at least for TS global costs, that is, performance difference between blocks in which the participant has to switch between tasks and blocks with only single tasks. There are thus counterexamples in which correlations with processing speed, even though significant, are of moderate entity, and cannot fully account for age-related effects (cf., Salthouse et al., 1998).

The speed of processing theory proposes two mechanisms to be responsible for age-related slowing as a consequence of a lower speed of processing (Salthouse, 1996): (i) the limited time mechanism, according to which early operations may take too long and therefore leave no time for later, possibly more complex operations; (ii) the simultaneity mechanism, that is, the assumption that products of lower level computations should be concurrently available for later processing. Despite these assumptions appear to be crucial on a theoretical ground, empirical testing is still scarce. To better characterize the relationship of speed of processing with TS performance, we used a cuing paradigm in which the cue-to-target interval (CTI) was varied across blocks, giving the participants more or less time to reconfigure or inhibit a previously active task-set. If limited time and simultaneity mechanisms were to operate, we should expect looser correlations with a longer CTI: by having more time to prepare for the task at hand, the impact of speed of processing on the performance should indeed be reduced. On the other hand, if mixing and switch costs do not depend on limited time mechanisms, we should observe no differences in correlations with processing speed across CTI conditions. We therefore built linear models for the two CTI conditions, always including a measure of processing speed as one of the predictors, to test whether the impact of such and other variables is actually higher when temporal constraints are stricter.

Other variables included in the models were chosen to investigate the contribution of other factors that are likely to play a major role in age-related cognitive decline. In particular, the construct of cognitive reserve (CR) has gained popularity during the last two decades (Stern, 2002, 2009; Valenzuela and Sachdev, 2006), with a number of studies demonstrating that the capacity of the cognitive system to cope with aging is also a function of previous life experiences. It is fair to say that early works mainly linked cognitive reserve to educational level, as the construct of cognitive reserve itself stems from those studies demonstrating a negative relationship between education and the severity of Alzheimer’s Disease symptoms (Katzman et al., 1988; Stern et al., 1992; Katzman, 1993). Nonetheless, life experiences other than education are also often considered to mediate age-related decline in older adults, mainly concerning occupational attainment (Satz et al., 1993; Stern et al., 1994; Garibotto et al., 2008) and leisure time activities (e.g., Wilson et al., 2002).

Even though it is plausible that education, being a relatively early event in life, plays a major role on the neural and psychological development of an individual, testing different dimensions of the CR construct becomes crucial for a better development of clinical tools and theoretical understanding of the aging process. For this reason, we chose to use the Cognitive Reserve Index questionnaire (CRIq) as a measure of cognitive reserve (Nucci et al., 2012): this tool provides both an overall measure of cognitive reserve and subscales related to each of the three dimensions cited above (education, occupational attainment, leisure time). In this way, it was possible to test the role for each of the relevant activities in a standardized manner. Even though CR and speed of processing are most likely to be involved in age-related differences when it comes to TS, there are a number of other variables that have been described as important in the EF literature. As depression is fairly frequently found among older adults (e.g., Beekman et al., 1999; Noël et al., 2004), and has been associated with reduced cognitive control (e.g., Meiran et al., 2011; Vallesi et al., 2015), Beck Depression Inventory (BDI) (Beck et al., 1961) scores were used as one of the regressors in the analyses. Finally, mild cognitive impairment and dementia have been found to affect TS performance on both mixing and switch costs (e.g., Belleville et al., 2008; Schmitter-Edgecombe and Sanders, 2009). We therefore chose to administer the Montréal Cognitive Assessment (MoCA, Nasreddine et al., 2005), as a measure of general cognitive integrity, and used its scores as another regressor in our analyses. In summary, building such large models allowed us to test and control for a variety of factors that might selectively affect TS performance in aging, taking into consideration possible confounds with speed of processing effects. Moreover, it was also possible to assess an important aspect of the processing speed theory, which is not much tested in the TS literature, namely the assumption that speed of processing is particularly relevant under high temporal constraints (e.g., Wingfield et al., 1985): in the present case, this was done by varying CTI across blocks.

## Materials and Methods

### Participants

A total of 98 volunteers (45 female), ranging from 21 to 79 years of age, were recruited through Internet and flyers. The whole experiment took place in a single session of 1.5 h at the Department of Neuroscience in Padova. Before starting the experiment, participants read and signed a consent form specifying the aim of the study and possible risks; another form was then presented in which they declared whether they had previous or current history of neurological or psychiatric problems, whether they had taken drugs or alcohol in the last 24 h and whether they had normal or corrected-to-normal vision.

### Test Description

All volunteers were administered 5 paper-and-pencil tests, and two computerized tasks: a TS paradigm and a Sustained Attention Reaction Time (SART) paradigm (not reported here)^1^. The sequence was fixed except that the computerized tasks were counterbalanced depending on the participant’s number (see Computerized Tasks). The order was: Edinburgh Handedness Inventory, Montreal Cognitive Assessment (MoCA), computerized tasks (counterbalanced), Symbol Digit Modalities Test (SDMT) and the BDI. A final questionnaire, the CRIq was administered only to those participants above 30 years of age, since the test is based on life-long experiences and younger individuals tend to systematically show lower scores just due to their relatively young age. Descriptive statistics of the sample are reported in **Table 1**, which shows participants divided into 3 age groups (21–30, 31–60, >60 years old), as commonly done in the aging literature (e.g., Charles et al., 2003; Bialystok et al., 2005).

**Table 1:** Average demographic data, scores and standard deviations (in parenthesis) for each experimental group.

	Younger adults (21–30 years old)	Middle aged (31–60 years old)	Older adults (>60 years old)
N	25	44	23
Females (%)	48	41	30.4
Age	25.3 (2.8)	46.6 (8.8)	69.6 (5.1)
Years of education	15.1 (2.8)	15.5 (3.9)	15.2 (2.7)
MoCA	28 (1.3)	27.9 (1.8)	26.7 (1.5)
SDMT	54.6 (9.6)	52.8 (11.8)	40.3 (9.1)
BDI	4 (3.3)	2.82 (2.6)	5.13 (3.4)
CRI-S	–	111.8 (14.2)	121.3 (9.4)
CRI-L	–	106.4 (14.3)	122.8 (15.6)
CRI-TL	–	121 (15.9)	119.8 (17.3)
CRI-Tot	–	117.3 (13.7)	128.2 (14.2)

*The performed tests were Montreal Cognitive Assessment (MoCA), Symbol Digit Modalities Test (SDMT), Beck Depression Inventory (BDI), Cognitive Reserve Index for Education (CRI-S), Working Activity (CRI-L), Leisure Time (TL), and total score (CRI-tot).*

### Computerized Tasks

Two computerized tasks were implemented on E-prime and then administered to each participant: a TS paradigm and a SART paradigm. The order of administration was counterbalanced according to demographic variables such as age range (in decades), gender and years of education (in 3 ranges): those sharing these features were pseudo-randomly assigned to one of four possible sequences to obtain a roughly similar number of participants per counterbalancing order in each population layer. The TS paradigm was indeed divided into two separate blocks with short or long CTIs. Task switching blocks were always performed consecutively, and the order of presentation was counterbalanced; TS blocks could be either preceded or followed by SART thus leading to the four possible aforementioned sequences. For data collection, we used a Dell laptop computer (Intel core i5-3320M CPU; 4 GB of RAM) with Windows 7 OS. Stimuli were presented on a 15-inch color monitor with a white background. Participants performed every task with a distance of about 50 cm from the screen.

### Task Switching Test

Each run of the TS paradigm was divided in 5 blocks: at the beginning participants had to perform two pure blocks consecutively, then the mixed block, finally two pure blocks again presented in reverse order with respect to the first two blocks in order to control for practice and fatigue effects. The two runs differed in CTI, that is, either 100 or 1200 ms (short and long CTI, respectively). The stimuli used were the letters A and E presented above or below a fixation cross.

In the pure blocks participants were asked to indicate either the identity of the letter at hand (verbal task) or its spatial location (spatial task): this was accomplished by pressing F or K on the keyboard, which were labeled as 1 or 2. In this way, the same keys were used for both the verbal and the spatial task, and the stimulus-response mapping was fixed across participants: F (labeled as 1) was to be pressed for A in the letter task, and when the letter appeared above the fixation cross in the spatial task; K (labeled as 2) had to be pressed for E, or when the letter was below the fixation cross depending on the task. Pure blocks began with instructions and 4 practice trials: at the end of practice participants were free to decide whether to start the test phase, or keep on with 4 extra practice trials. During practice, the experimenter monitored the responses and made sure that the participant understood the task to be performed. Moreover, during practice participants were also provided with a feedback indicating accuracy, and whether the response took too long: responses over 2000 ms were considered as non-responses. The practice phase with the pure block was repeated by 22 participants (on average, 4.5 extra trials each). The test phase consisted in 16 trials for each pure block: therefore, at each CTI, 32 trials were collected for each task, holding a total of 64 pure trials per CTI condition. In the mixed block a similar procedure was adopted: this time 16 practice trials had to be performed, always with a feedback; once again, participants were left free to decide whether to begin the test phase or to have another practice run: 24 subjects needed at least one extra practice (on average, 23.3 extra trials each).

**FIGURE 1:**
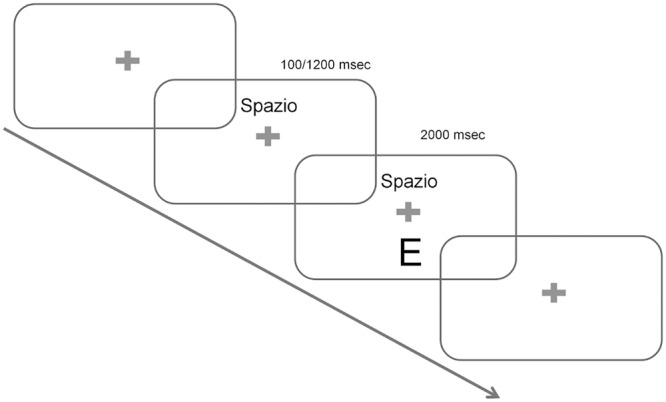
Experimental Paradigm. Each trial began with a fixation cross. Cues were then presented indicating the task at hand. The words “Spazio” and “Lettere” (Italian for “space” and “letters”, respectively) were used and stayed on the screen for the whole duration of the trial. The target appeared either after 100 or 1200 ms depending on the block (long and short Cue-Target-Interval, CTI, conditions), and stayed on the screen for 2000 ms. Finally, an Inter-trial-interval (ITI) varied randomly between 500 and 1000 ms and a new trial began.

The test consisted of 64 trials. **Figure 1** provides a graphical example of the procedure. At the beginning of each trial a cue indicating the task at hand was presented on the screen for 100 or 1200 ms depending on the run: the cues were transparent rather than arbitrary with respect to the task to be performed, as they were the words SPAZIO (Italian word for “space”) or LETTERA (Italian for “letter”). After this interval, the target stimulus appeared on the screen with the cue still present in its position (to minimize memory demands) and the participant had 2 s to provide a response. After this deadline passed by, both the target and the cue disappeared for a variable inter-trial interval ranging between 500 and 1000 ms, and a new trial began. The procedures described in this study were approved by the Comitato Etico per la Sperimentazione “Azienda Ospedaliera di Padova”.

## Data Analysis

The first trial of each mixed block was not considered in the analysis to avoid confounds with starting costs (Altmann, 2007). Five participants were excluded for excessive inaccuracy: to determine a threshold, a binomial analysis was carried out spotting those individuals responding at chance level (<60.8% correct) on the task switching conditions. After this, error and post-error trials were removed (7.8% of the total) (Rabbitt, 1966; Falkenstein et al., 2000). At this point, the distribution of RT data was analyzed to ensure normality: the distributions for each condition showed a skewness above 1; for this reason we transformed the data with a logarithmic function (Ratcliff, 1993; Whelan, 2008). The natural logarithm was used for this transformation: after the transformation, all the distributions survived to Shapiro–Wilk test for sphericity. Finally, outlier trials were determined for each participant in each condition (i.e., pure block, repeat trials, switch trials for each CTI): a cutoff value of two standard deviations above/below the mean was used (3.8% of the total trials were excluded). Analyses were conducted using R, version 3.4.0. Mixing cost and switch cost were calculated for both RTs and accuracy as in the literature (Roger and Monsell, 1995): Mixing cost = Performance difference between Repeat switching block trials and Pure block trials; Switch cost = Performance difference between Switch and Repeat switching block trials.

A 2 × 3 mixed ANOVA was performed for each cost, with CTI as the within-subject factor and group (21–30, 31–60, >60 years old groups) as the between subjects factor. Separate ANOVAs were run for errors and RTs. For *post hoc* analyses, a Tukey’s HSD test was used. Further, multiple regressions were performed on raw data for mixing and switch cost at different CTIs. Predictors were: age, MoCA score, years of education, BDI, and SDMT score. The latter was intended as a proxy of speed of processing. The same procedure was then used to build linear models excluding the youngest group (30 years of age or below): in this case years of education were not included in the models and were replaced with sub-scales and total score of the CRIq.

## Results

### ANOVAs

#### Mixing Cost

**FIGURE 2:**
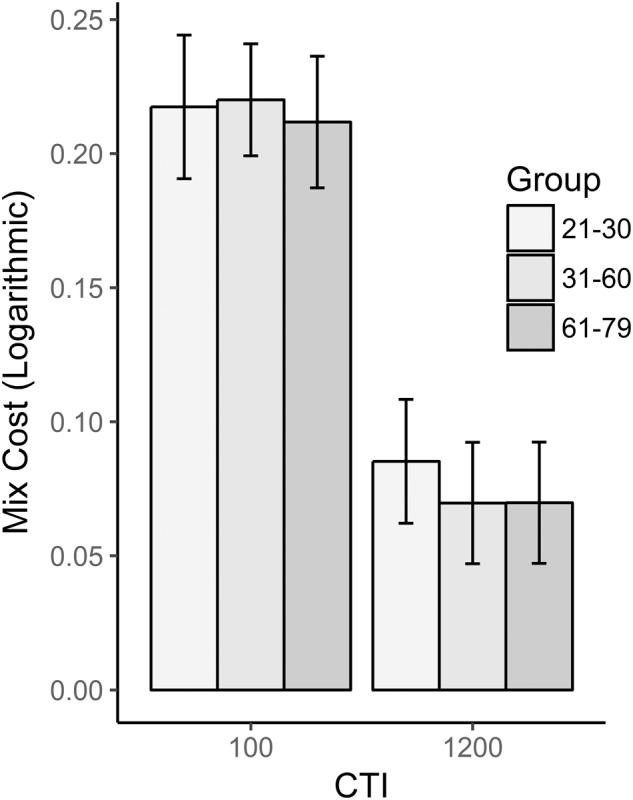
Mix cost as a function of group and CTI. RTs were logarithmically transformed before calculating the mix cost.

Mean RTs and accuracy for each group and CTI are reported in **Table 2** and **Figure 2**. Mixing cost in RTs decreased when more time to prepare was given to the participants, as evident from the main effect of CTI [*F*(1,90) = 261.57, *p* < 0.001, $\eta_{p}^{2}$ = 0.744]. This effect was constant across groups as no interaction emerged between Group and CTI [*F*(2,90) = 0.27, *p* = 0.76, $\eta_{p}^{2}$ = 0.008]. The absence of a main effect for Group [*F*(2,90) = 0.4, *p* = 0.67, $\eta_{p}^{2}$ = 0.006] indicated that the mixing cost did not significantly change with age. As far as the error analysis was concerned, results were substantially similar to those reported for RTs: once again, there was a main effect of CTI [*F*(1,92) = 6.94, *p* = 0.01, $\eta_{p}^{2}$ = 0.072] such that a longer time between cue and target was useful to correctly complete the task; no differences emerged between groups [*F*(2,92) = 0.785, *p* = 0.459, $\eta_{p}^{2}$ = 0.017] and no interaction was found between group and CTI [*F*(2,92) = 1.831, *p* = 0.166, $\eta_{p}^{2}$ = 0.039].

**Table 2:** Multiple linear regressions with mix cost at 100 and 1200 ms CTI as DVs.

	100 ms			1200 ms		
Predictor	Beta	Standard error	*P*-value	Beta	Standard error	*P*-value
Intercep	0	0.09	1	0	0.1	1
Age	–0.154	0.11	0.186	–0.237	0.12	0.055
Edu	–0.074	0.1	0.457	0.088	0.10	0.397
MoCA	–0.103	0.1	0.335	–0.221	0.11	0.051
BDI	0.024	0.1	0.809	–0.177	0.1	0.088
SDMT	–0.434	0.12	>0.001 ^∗∗∗^	–0.220	0.13	0.081
	Adjusted *R*^2^ = 0.149, *F*(5,87) = 4.217, *p* = 0.002			Adjusted *R*^2^ = 0.065, *F*(5,87) = 2.282, *p* = 0.053		

*Predictor variables include age, years of education, Montreal Cognitive Assesment, Beck depression inventary and Symbol-digit modalities test scores. Both dependent and independent variables have been standardized.*

#### Switch Cost

**FIGURE 3:**
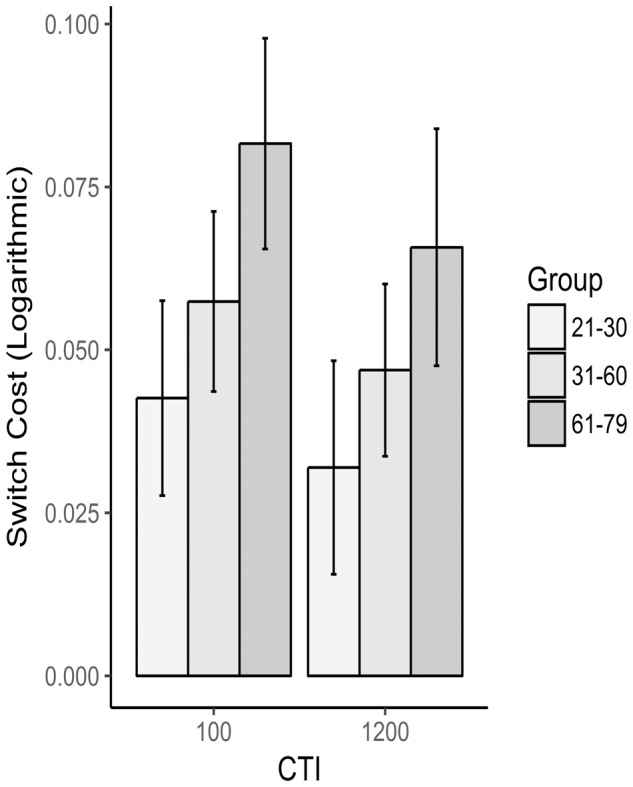
Switch cost as a function of group and CTI. RTs were logarithmically transformed before calculating the switch cost.

Mean RTs and accuracy for each group and CTI are reported in **Table 3** and **Figure 3**. As for the mixing cost, a main effect of CTI [*F*(1,90) = 5.32, *p* = 0.02, $\eta_{p}^{2}$ = 0.055] was found on the RT switch cost, consistent with the literature on task switching. Critically, however, also a main effect of group reached significance [*F*(2,90) = 7.24, *p* = 0.001, $\eta_{p}^{2}$ = 0.139]; Tukey’s pairwise comparisons indicated that the oldest group showed higher switch costs than the youngest (*p* < 0.001) and than the middle-age groups (*p* = 0.036). No differences emerged between the latter two groups (*p* = 0.19). Finally, CTI x Group interaction was well far from significance [*F*(2,90) = 0.1, *p* = 0.9, $\eta_{p}^{2}$ = 0.002], meaning that no group gained particular advantage from a higher preparation time in terms of switch costs. As far as errors were concerned, no significant effect was found either for CTI [*F*(1,92) = 0.355, *p* = 0.553, $\eta_{p}^{2}$= 0.004] or Group [*F*(2,92) = 2.866, *p* = 0.062, $\eta_{p}^{2}$ = 0.06].

**Table 3:** Multiple linear regressions with switch cost at 100 and 1200 ms CTI as DVs.

	100 ms			1200 ms		
Predictor	Beta	Standard error	*P*-value	Beta	Standard error	*P*-value
Intercept	0	0.09	1	0	0.09	1
Age	0.309	0.11	0.004 **	0.23	0.11	0.047 *
Edu	–0.221	0.09	0.016 *	0.001	0.1	0.989
MoCA	–0.065	0.09	0.506	–0.137	0.11	0.193
BDI	0.053	0.09	0.554	–0.102	0.1	0.291
SDMT	–0.224	0.11	0.0406 *	–0.229	0.12	0.053
	Adjusted *R*^2^ = 0.304, *F*(5,87) = 9.027, *p* < 0.001			Adjusted *R*^2^ = 0.1767, *F*(5,87) = 4.948, *p* < 0.001		

*Predictor variables include age, years of education, Montreal Cognitive Assesment, Beck depression inventary and Symbol-digit modalities test scores. Both dependent and independent variables have been standardized.*

### Linear Models

In order to test for a predictive role of speed of processing, cognitive reserve, mood, general cognitive integrity and age, 8 linear models were built: 4 including the whole sample, and 4 on a sub-population of older and middle-aged individuals only. Beside the sample, the first set of linear models differed from the latter in the measures of CR: as the CRIq was not administered to the youngest group, education was the only CR index in the whole-sample models. Results are summarized in the tables below (**Tables 2–5**). In general, nearly all models significantly predicted TS performance, but the amount of explained variance was consistently higher for the switch cost. This may be in line with the ANOVA’s results, indicating a main effect of group for switch cost only. As SDMT scores were negatively related with TS performance according to the models, it is conceivable that the older group, with lower scores on SDMT, showed higher switch costs. Also in line with ANOVAs is the drop of predictive power for both costs when the participants had more time to prepare: once again, as we observe that speed of processing is the most powerful predictor, then it is expected that models’ significance drops when time constraints are looser. Even though SDMT performance did not always correlate significantly with TS costs in all the conditions, it is to be noted that it did when correlated alone. The reason for this may be linked to shared variance between age and SDMT scores: correlation between these two variables was fairly high (*r*(91) = -0.53, *p* < 0.001, CI 95% [-0.66, -0.362]).

**Table 4:** Multiple linear regressions with mix cost at 100 and 1200 ms CTI as DV.

	100 ms			1200 ms		
Predictor	Beta	Standard error	*p*-value	Beta	Standard error	*p*-value
Intercept	0	1.12	1	0	0.12	1
Age	–0.092	0.18	0.616	0.057	0.19	0.763
CRI.s	0.021	0.13	0.87	0.056	0.13	0.676
CRI.l	–0.252	0.15	0.092	–0.167	0.15	0.28
CRI.tl	–0.036	0.12	0.766	–0.192	0.13	0.134
MoCA	–0.117	0.13	0.362	–0.263	0.13	0.052
BDI	0.118	0.12	0.336	–0.249	0.13	0.054
SDMT	–0.448	0.15	0.004 ^∗∗^	–0.0903	0.16	0.564
	Adjusted *R*-squared: 0.146, *F*(7,60) = 2.641, *p* = 0.019			Adjusted *R*-squared: 0.075, *F*(7,60) = 1.774, *p* = 0.109		

*Young adults excluded. Predictor variables include age, years of education, Montreal Cognitive Assesment, Beck depression inventary and Symbol-digit modalities test scores. Both dependent and independent variables have been standardized.*

**Table 5:** Multiple linear regressions with switch cost at 100 and 1200 ms CTI as DV.

	100 ms			1200 ms		
Predictor	Beta	Standard error	*P*-value	Beta	Standard error	*p*-value
Intercept	0	0.09	1	0	0.11	1
Age	0.294	0.16	0.064	0.098	0.18	0.595
CRI.s	–0.308	–0.11	0.007 ^∗∗^	0.106	0.13	0.418
CRI.l	0.052	0.13	0.681	0.084	0.15	0.575
CRI.tl	0.168	0.1	0.114	–0.08	0.12	0.518
MoCA	–0.068	–0.11	0.535	–0.171	0.13	0.189
BDI	0.067	0.1	0.522	–0.022	0.12	0.862
SDMT	–0.299	–0.13	0.023 ^∗^	–0.225	0.15	0.144
	Adjusted *R*-squared: 0.371, *F*(7,60) = 6.644, *p* < 0.001			Adjusted *R*-squared: 0.123, *F*(7,60) = 2.34, *p* = 0.035		

*Young adults excluded. Predictor variables include age, years of education, Montreal Cognitive Assesment, Beck depression inventary and Symbol-digit modalities test scores. Both dependent and independent variables have been standardized.*

Finally, education was found to negatively correlate with switch cost in the short CTI condition, whereas the mixing cost did not seem to be affected at all. Under all aspects cited above, models built excluding the youngest group were similar: the predictive power dropped at long CTIs, predictors accounted for more variance in the case of the switch cost, even though SDMT scores correlated significantly both with switch and mixing costs when considered alone. Furthermore, the subscale of CRIq for education, was still a valuable predictor for switch cost under high temporal constraints (i.e., short CTI). However, no other cognitive reserve variable had an impact on TS performance.

## Discussion

The aim of this study was twofold: on the one hand, the influence on TS performance of a number of variables related to cognitive aging was tested. To the best of our knowledge, despite a vast literature on TS and aging, no previous study had considered at the same time the role of both potentially protective and adverse factors in this frame. On the other hand, some aspects of processing speed theory have often been underestimated. Despite the theorization of a processing stream in which products of low level operations should remain available for later processing in order to successfully perform cognitive tasks (Salthouse, 1996), few studies were concerned with testing the role of speed of processing under different temporal constraints. There is consistent evidence that cognitive aging may be partially attributable to a decline in speed of processing (Salthouse, 1996). However, high variability in the elderly population suggests that multiple factors, including life-experience, may play an important role in shaping decline of cognitive functions in aging (Stern, 2002, 2009; Vallesi, 2016).

In order to better characterize these aspects in the executive domain, we implemented linear models using classical TS indices as dependent variables. Speed of processing, as operationalized with SDMT performance, was found to be consistently related to both mixing and switch costs, with a tendency to account for more variance in the latter case. In general, significance of the models was higher when the participants had little time to prepare (i.e., shorter CTI of 100 ms). This effect was driven by a drop of predictive power of SDMT scores and age: when temporal constraints were lower (i.e., longer CTI), it is likely that the capacity to quickly carry out simple operations becomes less relevant. This is in line with Salthouse’s (1996) processing speed theory. His model posits that age differences arise because the ability to carry out simple operations deteriorates over time. Performance in cognitive tasks is consequently disrupted since products of low level-operations are not available when successive, higher order operations, have to be executed. These results replicate other studies in which temporal demands have been manipulated to test this possibility in other domains (Salthouse and Coon, 1993; Kliegl et al., 1994).

Furthermore, variations of SDMT weight at different CTIs implicate that the weight of the speed of processing factor varies as a function of cognitive demands (Salthouse, 1992). It is to be noted, however, that correlations between processing speed and TS measures were moderate, and do not fully account for between subjects variability, replicating previous results (Salthouse et al., 1998; Cepeda et al., 2001). On the other hand, age was a significant predictor for the switch cost, suggesting that age-related effects on the latter are partly independent from general slowing, consistent with the literature on EFs (Verhaeghen and Cerella, 2002; Verhaeghen et al., 2006; Bugg et al., 2007).

Summing up, it is important to recognize that, even though speed of processing measures account for a significant part of the variance in executive performance, age-related differences persist beyond this construct. On a more fine-grained analysis, the relevance of general slowing may interact with experimental manipulations such as time-constraints, an often underestimated aspect in aging studies.

The role of cognitive reserve (CR) was also tested in the linear models. Contrary to our expectations, most indices of CR did not mediate TS performance. The only exception was education, being it negatively related to switch cost in the shorter CTI condition. For the interpretation of this result it might be important to underline that education was also the only CR index to significantly correlate with SDMT performance [*r*(91) = 0.24, *p* = 0.02]. It is possible to speculate that a high education level protects against speed of processing decline, in line with the literature (Cabral et al., 2016; Ihle et al., 2016). This would also be consistent with biological accounts of processing speed linking this construct to white matter integrity (Kerchner et al., 2012; Haász et al., 2013; Kuznetsova et al., 2016); also the original conceptualization of brain reserve, and subsequently CR, began with studies observing the beneficial effects of high education on brain pathologies affecting white matter connectivity (Katzman et al., 1988; Katzman, 1993).

However, other CR measures did not show the same effect. It is to be noticed that results concerning the role of CR in age-related EFs decline are ambiguous. Contrary to other domains, a relevant number of studies failed to establish a link between life experiences and a better performance in EF tasks. For instance, Hindle et al. (2017) in a recent study reported no difference between high and low CR Parkinson Disease patients in tasks tapping EFs. However, a similar research of the same group, also on a PD sample, revealed that education promoted various cognitive functions including EFs (Hindle et al., 2014), a pattern compatible to our data. Also research on non-pathological aging population is inconsistent on this point. Cabral et al. (2016) found a relation between CR and EFs; however, CR was operationalized only with measures of education (years of education, school failure, school attendance), or strictly related to it (reading books, newspapers and number of languages spoken). When using a more complex CR questionnaire, correlations with EFs or related functions sometimes fail to emerge (Puccioni and Vallesi, 2012b; Léon et al., 2014; Arcara et al., 2017).

Coming to the results of the ANOVAs, the finding of comparable mixing cost across groups is at odds with previous literature, as most of the studies find an increase in older adults (Kray and Lindenberger, 2000; Meiran et al., 2001; Adrover-Roig and Barceló, 2010; Lawo et al., 2012). However, a number of authors also report results similar to our (DiGirolamo et al., 2001; Kray et al., 2002; West and Travers, 2008). Classically, the mixing cost has been proposed to reflect a differential load on WM between the pure and the mixed block (Los, 1996; Kray and Lindenberger, 2000): as two task representations have to be held in the second case, this might slow down performance. This view is consistent with the finding of increased mixing cost on non-cued paradigms, where the participant has to keep track of the sequence. Within this frame, an increased mixing cost in older adults is a direct consequence of WM decline in aging (Kray and Lindenberger, 2000); coherently, in non-cued paradigms older adults’ performance suffers the most (Kray et al., 2002).

However, several studies cast doubts on the role of WM in mixing cost. Parametrically varying the number of task-sets does not produce any larger cost (Kramer et al., 1999; Rubin and Meiran, 2005), nor increases age-related differences (Buchler et al., 2008). As for switch cost, mixing costs are present only with bivalent stimuli (Mayr, 2001; Rubin and Meiran, 2005), namely stimuli on which both tasks may be potentially executed (as it is the case in the present study); also, introducing bivalent stimuli in one task elicits cautious responding on the other tasks performed in the same block employing univalent stimuli (Woodward et al., 2003; Metzak et al., 2013), a phenomenon which is known as the bivalency effect and is especially present in younger adults (Rey-Mermet and Meier, 2015). Finally, Mayr (2001) demonstrated that without overlap between S-R representations, mixing costs are absent: when responses were mapped differently for each task, or stimuli could not cue the competing representation, a mixing cost failed to emerge. Taken together, these results suggest that the mixing cost arises because of task conflict (Rubin and Meiran, 2005), resulting from a bottom-up activation of the competing task set: if there is no bivalency, there is no competition; on the other hand, the mixing cost would arise only in situations in which the two task-set representations overlap to some degree. The proposal of a stimulus-driven nature of the mixing cost is in line with findings of reduced mixing costs after extensive practice (Kramer et al., 1999; Buchler et al., 2008), since practice would help to reduce ambiguity.

What follows from these data is that older adults would particularly benefit from experimental manipulations aimed at reducing task ambiguity. For instance, Hirsch et al. (2016) report of no age-differences in mixing cost when presenting univalent stimuli. It is also possible to lower task ambiguity through the employment of strategies aiding a correct representation retrieval. Inner speech disruption through articulatory suppression has been consistently found to increase mixing costs in adults (Emerson and Miyake, 2003; Miyake et al., 2004; Saeki and Saito, 2009). Moreover, several studies have found an interaction between age and articulatory suppression costs, or explicit verbalization benefits (Kray et al., 2008, 2010; Chevalier and Blaye, 2009): children’s and older adults’ performance benefited more from verbalization, and was more disrupted by articulatory suppression compared to young adults’ performance. Supposedly, inner speech should help to form a WM representation about the task to be performed (Baddeley, 1996), or solve task-set competition; consistent with this, cue transparency has been found to reduce mixing and switching costs (Mayr and Kliegl, 2000; Arbuthnott and Woodward, 2002; Miyake et al., 2004; Logan and Schneider, 2006; Grange and Houghton, 2009): also in this case, ambiguity on the task to be performed next is lowered. In the present experiment transparent cues for the implementation of the TS paradigm were chosen. Indeed, the use of Italian words for “space” and “letter” to cue the upcoming task was likely to considerably reduce the competition in task selection. In other kinds of cueing paradigms, not only the cue does not solve ambiguity between tasks, but it also may be particularly detrimental, since it embeds an extra processing demand, as demonstrated by the literature on restart costs (Allport and Wylie, 2000; Poljac et al., 2009), namely the cost associated with cued repetition trials vs. uncued trials. It is therefore likely that older adults, who may have more difficulties in solving the ambiguity or forming correct WM representations, may benefit when an informative cue is used.

Coming to the switch cost, it is still debated whether it arises from an ongoing inhibition of a previously active task-set (Allport et al., 1994; Wylie and Allport, 2000), or it is due to a process of task-set reconfiguration (Roger and Monsell, 1995; Mayr and Kliegl, 2000; Monsell, 2003) or a mixture of the two (e.g., Tarantino et al., 2016; see Vandierendonck et al., 2010 for a review). However, its increase in older individuals had already been found in a number of studies using the cueing paradigm (DiGirolamo et al., 2001; Mayr, 2001; Meiran et al., 2001; Kray et al., 2002; Reimers and Maylor, 2005; West and Travers, 2008; Adrover-Roig and Barceló, 2010). There is a vast literature on differential cognitive strategies across the life-span indicating that older adults use a more bottom-up stimulus-triggered (reactive) strategy than top–down (proactive) cognitive control, consistent with the idea of an executive deficit in aging (Braver et al., 2005; Paxton et al., 2006). The use of cognitive control itself, as classically conceptualized by Norman and Shallice (1986), is required when routines do not suffice for the correct execution of the task: the use of a transparent cue may not aid much performance on switch trials, since extra information is needed (e.g., retrieval of different S-R rules); on repeat trials, however, a clear indication of the task to be performed may solve interference with the other task, thus facilitating task selection as proposed above. In other words, while using a transparent cue may be useful for repeat trials, such a beneficial effect is usually not found when a reconfiguration is needed (i.e., in switch trials). In individuals with lower speed of processing, this might prove particularly difficult; as a consequence, a bottom–up strategy may be implemented more often in older compared to younger adults. Since the endogenous component of the switch cost, as conceptualized by Monsell (2003), would be larger in older adults, a higher switch cost is expected. In the present case, not only switch cost was higher across CTIs for the older adults, but differences emerged more neatly at the short CTI, where speed of processing has been found to be a more robust predictor of TS performance.

## Conclusion

Even though the construct of cognitive reserve has gained popularity in the last decades, it is still controversial which specific life-experiences may actually protect from age-related decline in different cognitive functions (e.g., Puccioni and Vallesi, 2012b; Léon et al., 2014). The results of the present study showed that, among various proxies of cognitive reserve, only education is associated, under highly demanding time-constraints, with improved switch cost. On the other hand, common cause theories have been put forward to link cognitive decline in different functions to a general underlying cognitive domain. Among these, the speed of processing theory predicts poorer performance due to an inability to carry out simple operations at a high pace: if this was the case, we would expect speed of processing to play a more prominent role under high temporal constraints. Our results suggest that this is indeed the case: with short CTIs, our speed of processing measure predicted a significant portion of variance, while it did not with long CTIs. Finally, a pattern of increased switch cost and comparable mixing cost across the different age groups emerged in contrast with many the previous works. We suggest that the kind of cue employed (i.e., its degree of transparency) is the critical manipulation explaining the discrepancy of our results with previous studies. Despite it is conceivable that the choice of cue transparency may produce some consequences, these have not been systematically investigated so far, leaving a methodological issue in the TS literature that our study started to solve.

## Ethics Statement

This study was carried out in accordance with the Declaration of Helsinki. All volunteers gave their written informed consent prior to participation. The protocol was approved by the Bioethical Committee of the Azienda Ospedaliera di Padova.

## Author Contributions

AV designed the research project and designed the tasks. LM collected and analyzed the data. LM, CS, and AV wrote the manuscript.

## Conflict of Interest Statement

The authors declare that the research was conducted in the absence of any commercial or financial relationships that could be construed as a potential conflict of interest.

## Footnotes

1 This test was a modified version of that developed by Robertson et al. (1997). In the SART, participants were asked to respond to frequent ’go’ stimuli but maintain a readiness to withhold a response to rare and unpredictable no-go trials. In each trial, a single number appeared randomly in the center of the computer screen, and remained for 250 ms, before being substituted by a fixation cross that lasted for 900 ms. Participants had to press the “B” key on the keyboard if the stimulus presented was any number except the number “3”. The items were subdivided into a practice session of 18 trials (including 2 no-go trials) and a test session of 225 trials (including 25 no-go trials). The results of this task will be reported in future works as it falls outside the scope of the current study.
